# Vegfr2-luc转基因子代小鼠的鉴定

**DOI:** 10.3779/j.issn.1009-3419.2011.05.02

**Published:** 2011-05-20

**Authors:** 伟强 王, 红雨 刘, 志涛 宋, 玉丽 王, 竞 王, 颖 李, 永文 李, 岷 王, 军 陈, 清华 周

**Affiliations:** 1 300052 天津，天津医科大学总医院，天津市肺癌研究所，天津市肺癌转移与肿瘤微环境实验室 Tianjin Key Laboratory of Lung Cancer Metastasis and Tumor Microenviroment, Lung Cancer Institute, Tianjin Medical University General Hospital, Tianjin 300052, China; 2 300052 天津，天津医科大学总医院胸部肿瘤中心 Department of Toracic Tumor Center, General Hospital of Tianjin Medical University

**Keywords:** 动物模型, 血管新生, 荧光素酶报告基因, Animal model, Angiogenesis, Luciferase

## Abstract

**背景与目的:**

Vegfr2-luc转基因小鼠体内血管内皮生长因子受体2（vascular endothelial growth factor receptor 2, VEGFR2）的表达可以驱动荧光素酶报告基因（luciferase, luc）的表达，是活体动物水平实时监测血管生成情况的有利工具。本研究旨在对子代Vegfr2-luc转基因小鼠进行鉴定，以确定能否用于血管生成研究。

**方法:**

PCR检测新生小鼠基因组内*luc*基因；利用活体成像技术观察新生Vegfr2-luc转基因小鼠生长发育过程中以及皮肤伤口修复过程中*luc*基因表达水平的变化情况；荧光素酶报告基因检测试剂盒检测成年（8周龄）转基因小鼠各脏器荧光素酶的活性和Real-time PCR检测各器官VEGFR2 mRNA的表达水平。

**结果:**

PCR结果显示50%（56/112）的新生小鼠携带*luc*基因。活体成像结果显示随着Vegfr2-luc转基因小鼠发育成熟，luc表达量逐渐降低（*P* < 0.001）；在皮肤伤口修复过程中，伤口处luc表达水平先增强后降低（*P* < 0.001）。雌性成年转基因小鼠各脏器VEGFR2 mRNA的表达水平与荧光素酶活性呈正相关（*r*=0.948, *P* < 0.001）。将睾丸组织除外，雄性成年转基因小鼠各脏器VEGFR2 mRNA的表达水平与荧光素酶活性同样呈正相关（*r*=0.836, *P* < 0.001）。

**结论:**

Vegfr2-luc转基因子代小鼠体内luc表达水平的变化可以反映VEGFR2的表达情况。

## Abstract (en)

**Background and objective:**

A transgenic mouse, Vegfr2-luc, in which a luciferase reporter (luc) is under control of the murine VEGFR2 promoter, can be used to track angiogenesis *in vivo*. The aim of this study is to identify the offspring of Vegfr2-luc transgenic mouse.

**Methods:**

Luc was detected with PCR in genomic DNA of the new-born mouse. Luc expression in the offspring of Vegfr2-luc transgenic mouse was monitored with IVIS *in vivo* imaging system during post-natal development. Wound-healing models of Vegfr2-luc transgenic mouse offspring were established and the expression of luc was monitored during the wound-healing process. Luc activity and VEGFR2 mRNA expression in different organs were detected with luc Assay System and Real-time PCR respectively.

**Results:**

PCR showed that 50% (56/112) of the offspring of Vegfr2-luc transgenic mouse carry luc. IVIS *in vivo* imaging results demonstrated that luc expression in Vegfr2-luc transgenic mouse dropped dramatically with age increase (*P* < 0.001) and luc expression in the wound first increased and then decreased during the wound-healing process (*P* < 0.001). Luc activity in female Vegfr2-luc transgenic mouse organs was positively correlated with VEGFR2 mRNA expression (*r*=0.948, *P* < 0.001). Except testis, luc activity in male Vegfr2-luc transgenic mouse organs was also positively correlated with VEGFR2 mRNA expression (*r*=0.836, *P* < 0.001).

**Conclusion:**

The offspring of Vegfr2-luc transgenic mouse is applicable to tracking angiogenesis *in vivo*.

血管内皮生长因子（vascular endothelial growth factor, VEGF）家族有三个受体，即VEGFR1、VEGFR2和VEGFR3。VEGFR1募集造血干细胞并促进单个核细胞和巨噬细胞迁移；VEGFR2参与调节血管内皮细胞的功能；VEGFR3参与调节淋巴管内皮细胞的功能。VEGFR2介导的细胞内信号级联反应促进血管内皮细胞增生、迁移、存活和通透性增加，从而促进血管新生，参与胚胎发育、损伤修复和许多疾病的发展进程^[1]^。最近的研究^[2]^显示，VEGFR2参与结肠炎向结肠癌的演变过程，长期慢性炎症导致肠上皮细胞表达VEGFR2增多。特异性阻断VEGF和VEGFR2之间相互作用的人源抗体r84对肺癌动物模型显示出良好的抗肿瘤活性^[3]^。如何阻断肿瘤组织的血管新生已经成为肿瘤治疗研究的热点之一，VEGFR2是抗血管新生重要的靶分子^[4]^。在活体内纵向研究肿瘤血管新生有很多限制，如为了观察肿瘤血管内皮细胞标记分子VEGFR2的表达情况，通常需要在不同的时间点处死小鼠取出肿瘤组织进行免疫组化检测，使研究的持续性和一致性不能得到保证。以非侵害性的方式在活体内追踪VEGFR2表达的变化情况将对抗肿瘤血管新生研究提供极大的便利。

美国Xenogen公司将luc报告基因“敲入”VEGFR2基因启动子下游，使VEGFR2的表达能够驱动luc的表达，然后将Vegfr2-luc基因片断导入小鼠染色体，成功制备Vegfr2-luc转基因小鼠，利用活体成像技术能以非侵害性的方式追踪Vegfr2-luc转基因小鼠体内VEGFR2的表达情况^[5]^。有两个研究小组^[6, 7]^应用Vegfr2-luc转基因小鼠分别对松弛素（relaxin）和接合纤维素的VEGF（fibrin-conjugated）的促血管新生作用进行了研究，表明Vegfr2-luc转基因小鼠是研究体内血管新生的良好工具。Angst等^[8]^应用Vegfr2-luc转基因小鼠制备了胰腺癌原位肿瘤模型，并使用IVIS活体成像系统实时检测了胰腺癌的血管新生情况。天津市肺癌研究所从美国Xenogen公司引进Vegfr2-luc转基因小鼠两对，进行繁育并鉴定，拟用于肺癌和其它肿瘤血管新生方面的研究。

## 1 材料与方法

### 1.1 材料

#### 1.1.1 主要仪器设备

IVC设施（智能型）购自苏州市冯氏实验动物设备有限公司；小动物活体成像系统（IVIS 200 Living Imaging System）购自美国XENOGEN公司；7900实时定量PCR（real-time PCR）仪购自美国ABI公司；连续光谱酶标仪购自美国Molecular Devices公司；凝胶成像系（ChemiDoc XRS System）购自美国Bio-Rad公司；台式微型离心机（Microfuge 18）购自美国Beckman Coulter公司；超净台购自中国Airtech公司；微量移液器（P-2.5, P-10, P-100, P-200, P-1000）购自德国Eppendorf公司；高压灭菌器HVP-50购自日本Hirayama公司。

#### 1.1.2 实验动物

Vegfr2-luc转基因小鼠，雄鼠为纯合（nu/nu），雌鼠为杂合（nu/+），购自美国Xenogen公司。

#### 1.1.3 试剂

总RNA提取试剂Tr izol Reagent购自美国Invitrogen公司；M-MLV逆转录酶、随机引物、RNase抑制剂、dNTP mix和荧光素酶检测试剂盒（Luciferase Assay System）购自美国Promega公司；DNA Marker DL2000购自日本TAKARA公司；荧光素（D-Luciferin Firefly）购自美国Xenogen公司；小鼠全价营养颗粒饲料购自维通利华公司；蛋白酶K购自北京天根公司；VEGFR2实时定量PCR引物、Goldview核酸染料购自北京赛百盛公司；琼脂糖粉购自美国Sigma公司；SYBR GREEN Master Mix和八联管购自美国ABI公司；BCA蛋白浓度测定试剂盒购自美国PIERCE公司；无RNase无菌包装枪头、枪头盒、EP管及PCR管购自美国AXYGEN公司；无水乙醇、异丙醇、三氯甲烷、EDTA、氯化钠等均为国产分析纯产品。

### 1.2 方法

#### 1.2.1 实验小鼠的饲养和管理

Vegfr2-luc转基因小鼠饲养在IVC设施内，每周饲喂、换饮、清洁2次。记录转基因小鼠每胎出生个数、成活率及生长发育情况。

#### 1.2.2 小鼠组织DNA提取

小鼠出生21天离乳分笼时剪取小鼠尾巴（约0.5 cm），加入600 μL TNES（10 mM Tris pH7.5, 400 mM NaCl, 100 mM EDTA, 0.6%SDS）和18 μL蛋白酶K（20 mg/mL）55 ℃孵育24 h；加入166.7 μL 6 M NaCl，剧烈震荡15 s后12, 000 rpm离心5 min；取出上清，加入等体积冰上预冷的95%乙醇，12, 000 rpm离心5 min；弃去上清，加入0.5 mL 70%乙醇漂洗，12, 000 rpm离心5 min；沉淀即为小鼠基因组DNA。弃去上清，待沉淀风干后加入200 μL灭菌去离子水溶解，测DNA浓度，置于-20 oC冰箱备用。

#### 1.2.3 PCR鉴定luc^+^小鼠

取小鼠基因组DNA 100 ng作为模板进行PCR反应。*Luc*基因引物序列：上游为5'-TGGATTCTAAAACGGATTACCAGGG -3'，下游为5'-CCAAAACAACAACGGCGGC-3'，扩增产物长度为1, 000 bp。内参β-globulin引物序列：上游为5’-CAGACGCCACTGTCGCTTT-3’，下游为5’-TGTCTTTGGAACTTTGTCTGCAA-3’，扩增产物长度为500 bp。PCR反应条件：94.5 ℃、40 S；58 ℃、1.5 h；72 ℃、1.5 h，共进行35个循环。取PCR产物10 μL在1%琼脂糖凝胶中电泳，凝胶成像仪上观察并成像。

#### 1.2.4 小鼠活体荧光成像

异氟烷吸入性麻醉Vegfr2-luc转基因小鼠，腹腔注射荧光素（luciferin）150 mg/kg，10 min后应用IVIS200活体成像系统进行成像，IVIS活体成像软件分析成像结果。分别对3、4、6、8、10和15周龄Vegfr2-luc转基因子代小鼠背侧位和腹侧位进行全身成像（每个时间点成像3只小鼠），曝光时间为20 s，为去除身体面积不同造成的偏差，取单位身体面积所发出的光子数为实验结果（p/s/cm^2^）。损伤修复模型建立后，每隔3天-4天对伤口成像一次，直到第21天损伤修复基本完成，曝光时间为1 s，伤口处荧光值的单位为p/s。

#### 1.2.5 皮肤损伤修复模型的建立

取3只8周龄雌性Vegfr2-luc转基因子代小鼠，将背部相应部位皮肤的毛发剪掉，然后用剪刀剪去一块直径约为8 mm的圆形全层皮肤（包括上皮层、真皮层和皮下组织）。

#### 1.2.6 小鼠各脏器组织蛋白中荧光素酶活性的测定

麻醉处死6只（雌雄各3只）8周龄转基因子代小鼠，取肺、心、肝、肾、肌肉、子宫和睾丸等脏器。从各脏器中取100 mg组织置于研钵中，加入液氮，将其研磨成精细粉末；加入PLB（passive lysis buffer），在液氮和37 ℃水浴中反复冻融3次，以充分裂解蛋白；12, 000 rpm离心4 min，收集上清，上清中即为组织蛋白。应用BCA法测定组织蛋白浓度。蛋白定量后按照荧光素酶检测试剂盒的说明检测各脏器组织蛋白中荧光素酶的相对活性。

#### 1.2.7 Real-time PCR检测各脏器组织中VEGFR2 mRNA的表达水平

取获取的各脏器组织100 mg，Trizol法提取总RNA，通过异丙醇沉淀法浓缩RNA，分光光度计定量，甲醛变性胶电泳检测总RNA质量。按照Promega逆转录试剂盒说明书进行操作合成cDNA。所用引物序列引自文献^[9]^，由赛百盛生物公司合成。VEGFR2引物序列如下：上游5’-GCCCTGCTGTGGTCTCACTAC-3’，下游5’-CAAAGCATTGCCCATTCGAT-3’；内参基因为小鼠Cyclophinin，引物序列为：上游5’-CAGACGCCACTGTCGCTTT-3’，下游5’-TGTCTTTGGAACTTTGTCTGCAA-3’；按照ABI SYBR Green Master Mix试剂盒说明书操作。

### 1.3 统计学处理

应用SPSS 16.0统计软件进行处理。计量资料用Mean±SD，组间比较采用单因素方差分析，相关分析采用直线相关。基因mRNA表达水平比较采用相对定量ΔΔCT值比较法进行，ΔΔCt=[Ct GI（未知样品）-Ct Cyclophinin（未知样品）]-[Ct GI（校正样品）-Ct Cyclophinin（校正样品）]，平均相对含量= 2^-averageΔΔCT^。

## 2 结果

### 2.1 转基因小鼠繁育状况及luc^+^新生小鼠的比例

转基因小鼠在IVC设施内生长繁育良好，每胎出生6只-10只不等，截止发稿时已繁育3代，共13胎，112只，成活率为100%。如图 1所示*luc*基因是1, 000 bp，内参β-globulin是500 bp。Luc^+^新生小鼠的比例为50%（56/112）。

**1:**
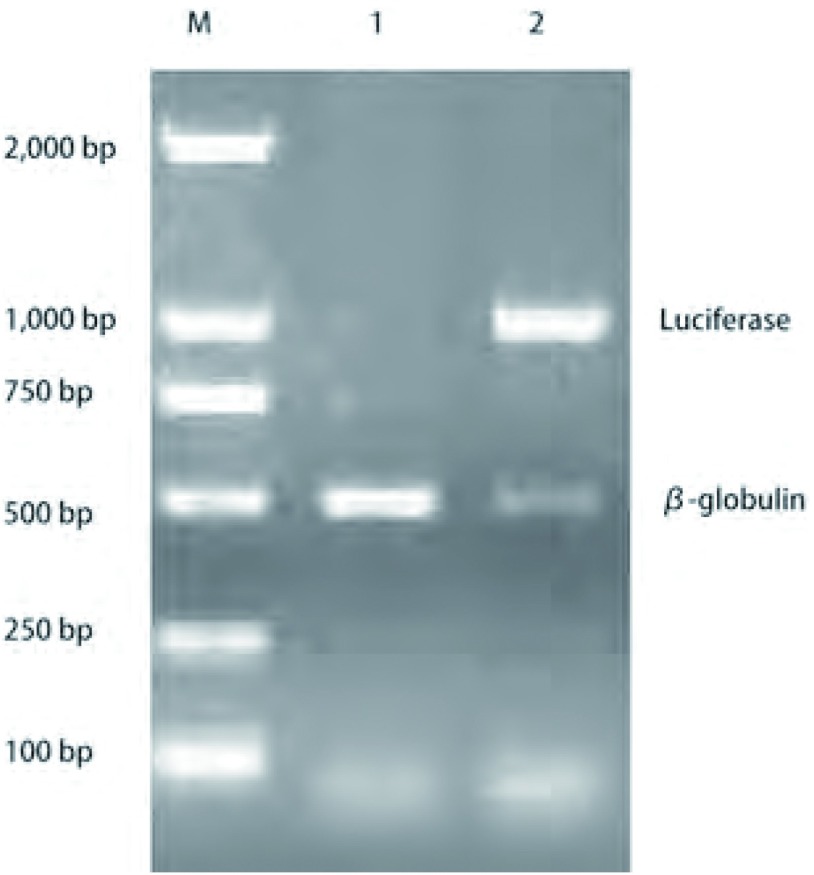
PCR鉴定luc报告基因。M：DNA marker；1：luc-小鼠；2：luc^+^小鼠 Detection of luc reporter gene DNA marker. 1: luc- mice; 2: luc^+^ mice

### 2.2 转基因小鼠发育过程中luc荧光值的变化情况

随着鼠龄增长，各组小鼠腹侧位和背侧位活体成像所获得的luc荧光值逐渐降低，不同周龄小鼠荧光值之间的差异有统计学意义（*P* < 0.001）；15周龄转基因小鼠与3周龄相比luc荧光值差异有统计学意义（*P* < 0.001），且已降至较低水平（图 2）。

**2:**
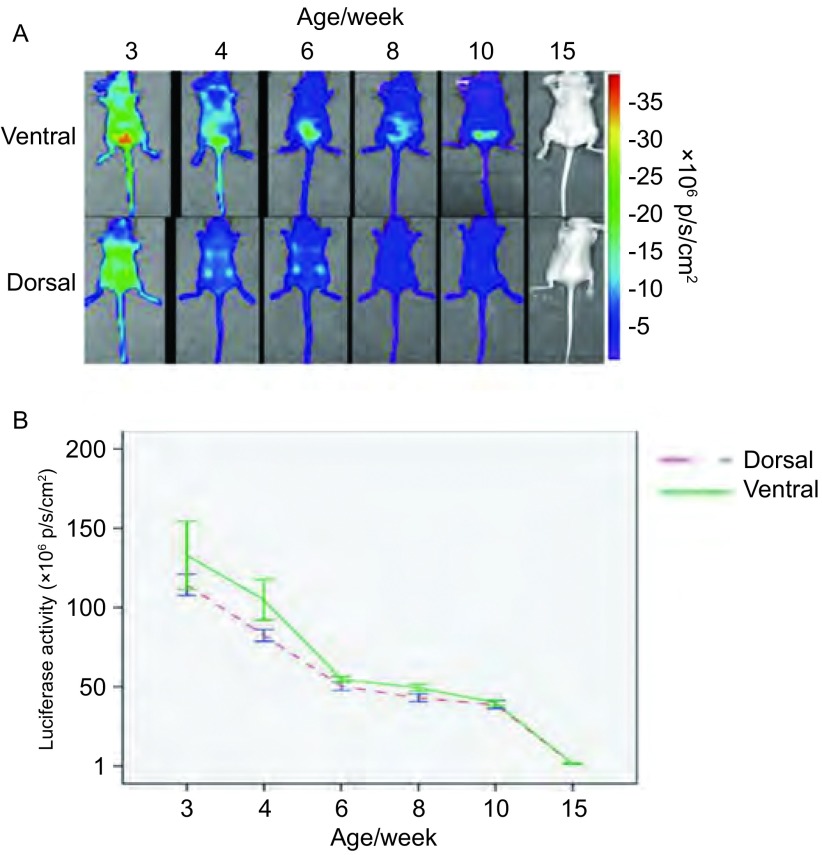
Vegfr2-luc转基因小鼠生长发育过程中luc的表达情况。A：雌性Vegfr2-luc转基因小鼠（*n* =3）分别在出生后的3、4、6、8、10和15周进行腹侧位和背侧位成像，成像的颜色越靠近标尺的上端，表明荧光信号越强；B：应用活体成像分析软件量化小鼠整个身体所发出的荧光值（p/s/cm^2^）。随着鼠龄增长，luc表达量逐渐降低（*P* < 0.001） Luc expression during postnatal development. A: Female Vegfr2- luc mice (*n* =3) at the ages of 3, 4, 6, 8, 10, and 15 weeks were imaged with IVIS in vivo living imaging system. Luciferase signal was collected from the dorsal and ventral sides of the mice. The color overlay on the image represents the photons per second emitted from the animal, as indicated by the color scales; B: Quantification of luc signal from the whole body with Living Image software (p/s/cm^2^). Luc expression in Vegfr2-luc transgenic mouse decreased with age (*P* < 0.001)

### 2.3 转基因小鼠各脏器luc活性和VEGFR2 mRNA的表达情况

雌性转基因小鼠各脏器荧光素酶活性从高到低的顺序依次为肺脏、子宫、肾脏、心脏、骨骼肌和肝脏，不同脏器luc活性的差异有统计学意义（*P* < 0.001）；VEGFR2 mRNA的表达水平从高到低的顺序依次为肺脏、子宫、肾脏、心脏、骨骼肌和肝脏，不同脏器VEGFR2 mRNA表达水平的差异有统计学意义（*P* < 0.001）（图 3）；各脏器荧光素酶活性和VEGFR2 mRNA的表达水平呈正相关（*r*=0.948, *P* < 0.001）。雄性转基因小鼠各脏器荧光素酶活性从高到低的顺序依次为肺脏、睾丸、肾脏、心脏、骨骼肌和肝脏，不同脏器luc活性的差异有统计学意义（*P* < 0.001）；VEGFR2 mRNA的表达水平从高到低的顺序依次为肺脏、肾脏、心脏、睾丸、骨骼肌和肝脏，不同脏器VEGFR2 mRNA表达水平的差异有统计学意义（*P* < 0.001）；将睾丸除外后，各脏器荧光素酶活性高低和VEGFR2 mRNA的表达水平呈正相关（*r*=0.836, *P* < 0.001）。

**3:**
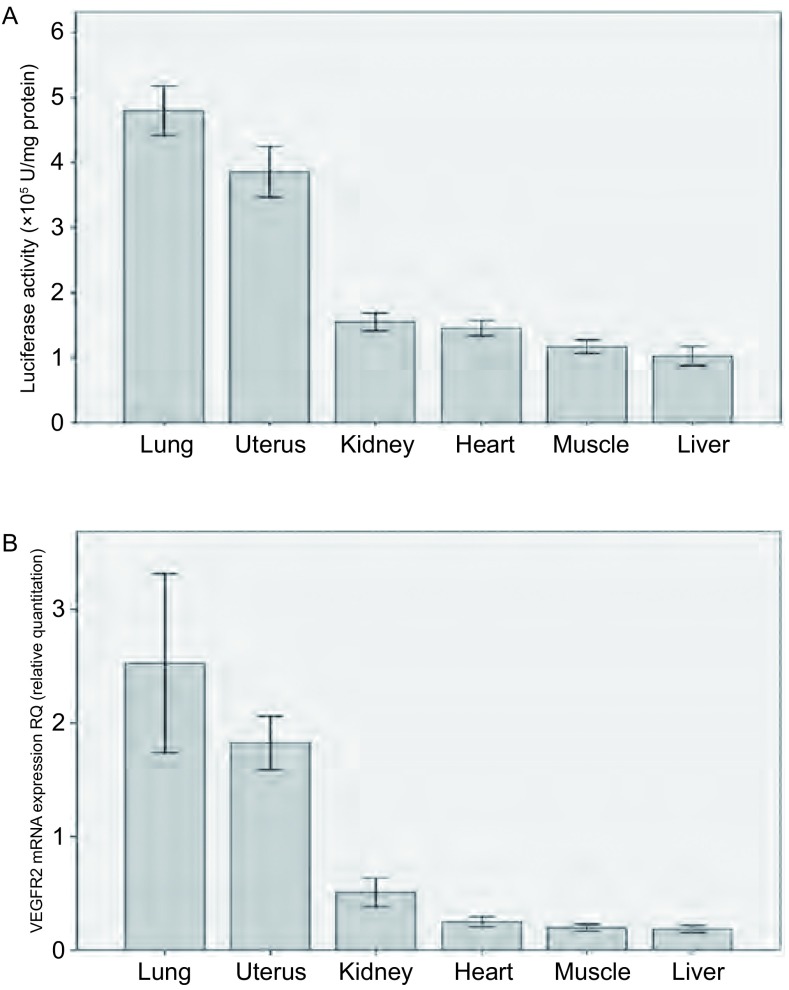
雌性8周龄Vegfr2-luc转基因小鼠（*n* =3）各脏器luc活性和VEGFR2 mRNA的表达情况。A：转基因小鼠各脏器组织蛋白的luc活性；B：Realtime PCR检测各脏器组织中VEGFR2 mRNA的表达情况。各脏器VEGFR2 mRNA的表达水平与荧光素酶活性存在相关性（*r* =0.948, *P* < 0.001） Vegfr2-luc expression in different tissues of 8-week female transgenic mice (*n* =3). A: Luc activity in different organs; B: Real-time PCR analysis of VEGFR2 mRNA expression in different organs. Luc activity correlated with VEGFR2 mRNA expression (*r* =0.948, *P* < 0.001)

### 2.4 皮肤损伤修复过程中伤口处luc荧光值的变化情况

转基因小鼠皮肤损伤修复模型刚刚建立时，活体成像没有检测到luc荧光值，随着伤口修复过程不断推进，luc荧光值不断增加，第7天-第10天时达到峰值。随着修复过程逐渐完成，luc荧光值逐渐降低，到第21天修复基本完成时，只能检测到少量luc荧光信号。不同时间点luc荧光值的差异有统计学意义（*P* < 0.001）（图 4）。

**4:**
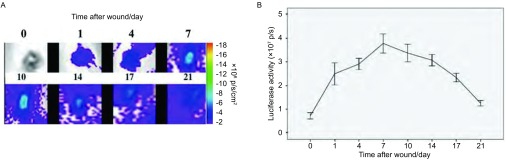
皮肤损伤修复过程中伤口处的luc表达情况。A：损伤修复过程中不同时间点的成像结果（*n*=3）。0天为损伤后立即成像，之后分别在损伤后的第1、4、7、10、14、17和21天进行成像；B：应用活体成像软件量化伤口处的荧光值（p/s）。伤口处luc荧光值先逐渐增强后逐渐减弱(*P* < 0.001) Luc expression during wound-healing. A: The wound-healing model mice were imaged at selected time points (*n* =3). The day 0 image was taken immediately after wounding, and subsequent images were taken on days 1, 4, 7, 10, 14, 17, and 21 after wounding. B: Quantification of luciferase expression (p/s) from the wound area with Living Image software. Luc expression first increased, and then decreased in the wound area (*P* < 0.001)

## 3 讨论

参考文献Vegfr2-luc转基因小鼠基因组内的VEGFR2是共显性等位基因，其中一条等位基因的表达必然伴随着另外一条等位基因的表达。*Luc*报告基因被插入到VEGFR2其中一条等位基因的第一个外显子，VEGFR2的表达会驱动*luc*报告基因的表达。因此，Vegfr2-luc转基因小鼠的VEGFR2等位基因位点是杂合子（luc/VEGFR2），理论上新生小鼠中携带*luc*报告基因的比例为50%。PCR检测新生小鼠DNA中的*luc*报告基因，新生小鼠中的50%携带luc报告基因，与理论值相符。

VEGF与其受体VEGFR2的相互作用对小鼠胚胎发育以及出生后体内的血管新生都起着至关重要的作用。小鼠出生后发育过程的早期阶段伴有大量血管新生，VEGFR2的表达也相应上调^[1]^。当小鼠发育成熟后，血管新生明显减少，VEGFR2的表达也降低。不同周龄Vegfr2-luc转基因小鼠的活体成像结果显示，发育早期小鼠luc报告基因的表达水平较高，随着小鼠成熟度增加，*luc*报告基因的表达逐渐减少，到6周龄以后，表达处于一个相对稳定的较低水平。Luc报告基因在成年雌性Vegfr2-luc转基因小鼠的肺脏和子宫的表达水平较高，在其它检测的脏器中的表达相对较低。成年雄性Vegfr2-luc转基因小鼠的肺脏和睾丸*luc*报告基因的表达水平较高，其它脏器表达水平较低。将雄鼠的睾丸组织除外，成年Vegfr2-luc转基因小鼠各器官组织中VEGFR2 mRNA的表达水平与luc报告基因的表达水平成正相关，这与XENOGEN公司发表的关于Vegfr2-luc转基因小鼠的文献^[5]^相一致。

通常将小鼠背部皮肤的全皮层（包括表皮、真皮和皮下组织）切除制备皮肤损伤模型，用于皮肤损伤修复过程或其它皮肤疾病的研究^[10]^。皮肤损伤诱导产生的过氧化氢（H_2_O_2_）能促进VEGF和VEGFR2表达增加，从而促进血管新生，有利于损伤修复^[11]^。与皮肤伤口修复的过程相一致，伤口处的血管新生会先增多后减少，直到修复完成。在Vegfr2-luc转基因小鼠损伤修复模型建立后的第1天即能检测到luc的表达，第7天-第10天时表达达到最高水平，第21天修复即将完成时仍有少量表达。这说明在转进因小鼠的损伤修复过程中，luc的表达伴随着损伤修复过程中的血管新生。

综上所述，新生Vegfr2-luc转基因小鼠体内luc报告基因的表达能够示踪体内VEGFR2的表达情况，是在动物水平非侵入性研究血管新生的有利工具。
